# Management of *Pseudomonas aeruginosa* pneumonia: one size does not fit all

**DOI:** 10.1186/cc13849

**Published:** 2014-04-29

**Authors:** Jordi Rello, Bárbara Borgatta, Leonel Lagunes

**Affiliations:** 1 Critical Care Department, Hospital Vall d’Hebron, Passeig de la Vall d’Hebron, 119-129, 08035 Barcelona, Spain; 2 CIBERES, Recinto Hospital Joan March, Carretera Soller Km 1207110, Mallorca, Bunyola, Illes Balears, Spain; 3 Vall d’Hebron Institute of Research, Passeig de la Vall d’Hebron, 119-129, 08035 Barcelona, Spain; 4 Universitat Autònoma de Barcelona, Plaça Cívica, Campus de la UAB,Sardañola del Vallés, 08193 Barcelona, Spain

## Abstract

In view of the mortality associated with *Pseudomonas aeruginosa* (PSA) ventilator-associated pneumonia (VAP) and the frequency of inadequate initial empiric therapy, recent findings underscore the need for a different management paradigm with effective anti-pseudomonal vaccines for prophylaxis of patients at risk. The association of virulence factors is a variable that splits PSA in two phenotypes, with the possibility of adjunctive immunomodulatory therapy for management of virulent strains. We comment on recent advances in and the state of the art of PSA-VAP management and discuss a new paradigm for tailored and optimal management.

## 

In the previous issue of *Critical Care*, Lu and colleagues [1] reported a visionary study assessing the distribution of *Pseudomonas aeruginosa* (PSA) serotypes in patients with ICU pneumonia and suggested differences in outcomes depending on serotypes. In this report, serotype O6 predominated, being associated with better clinical outcomes than serotype 011, which were frequently producing toxins secreted by the type III secretion system (TTSS). These findings have important implications for both clinical practice and future studies.

In an international study of over 1,200 ICUs in 75 countries, the risk of infections, including those due to *Pseudomonas* species, was found to increase with duration of ICU stay; in addition, infection was associated with an increased risk of mortality [2]. In 2014, at a time when multidrug-resistant clones are emerging and represent a strong risk of dissemination, we have much more information on *Pseudomonas* pneumonia management. We know that one effective agent is equivalent to two [3,4] but that initial combination followed by de-escalation improves survival by reducing the risk of delay in appropriate therapy. We know that resolution of episodes with appropriate therapy is similar to core pathogens [5] but that wrong initial therapy is associated with a resolution similar to that of methicillin-resistant *Staphylococcus aureus *[6].

Pulsed-field electrophoresis analysis performed in an ICU with a high prevalence of PSA identified the genotypes of more than 1,700 isolates [7]. Interestingly, the most frequently isolated clones were responsible for gut or skin colonization, in addition to respiratory colonization, but were only rarely associated with pneumonia. When ventilator-associated pneumonia (VAP) was present, most patients achieved clinical resolution without major consequences. On the other hand, non-related clones suggestive of prior colonization were associated with a very high mortality rate [7]. Most clonally related isolates caused gastric colonization before skin or respiratory tract colonization, suggesting an association with the tap water used in the administration of medication. These findings emphasize that different risk factors may be implicated depending on whether the clone is due to exogenous contamination or or as endogenous colonization from being a carrier. Therefore, conventional identification provided by the microbiology laboratory results is insufficient for assessing the patient and effective management.

Indeed, recent advances have demonstrated the importance of virulence factors in PSA infections. Although several different mechanisms such as quorum sensing and biofilm formation have been reported [8], the TTSS, encoded by PSA, has become one of the most important and widely studied virulence factors. After the microorganism has come into contact with the cell, the needle-like TTSS mechanism allows the bacteria to inject toxins directly into the cytoplasm of the host cell [9], evading direct recognition by the host’s immune system [10]. Recent studies suggest that failure to eradicate PSA in patients with VAP may be linked to TTSS. Patients infected with *Pseudomonas* sp*.* strains which express at least one type of TTSS protein (TTSS^+^) at the onset of VAP are more likely to have recovered at day 8 post-VAP, whereas eradication is achieved in patients with undetectable levels of TTSS proteins [11]. The transfer of our knowledge of the virulence factors to the clinical setting is crucial in order to evaluate the potential of virulence factor-directed therapies.

In view of the mortality associated with PSA-VAP [3,5,12] and the frequency of inadequate initial empiric therapy [13-15], these findings underscore the need for a different management paradigm with effective anti-pseudomonal vaccines for prophylaxis of patients at risk and the need for rapid diagnostic test methods and monoclonal-specific antibodies blocking virulence factors in patients with VAP.

We have also learned that association of virulence factors is a variable that splits *P. aeruginosa* in two phenotypes, with the possibility of adjunctive immunomodulatory therapy for management of virulent strains [16]. A combination of general risk factors and molecular diagnosis techniques may identify suitable candidates for intervention. As in invasive pneumococcal infections [17], further research is required to identify potential associations of comorbidities and serotypes as well as of serotypes and specific complications.

## Abbreviations

PSA: *Pseudomonas aeruginosa*; TTSS: Type III secretion system; VAP: Ventilator-associated pneumonia.

## Competing interests

JR has served on advisory boards or speakers bureau (or both) for Kenta Biotech (Zürich-Schlieren, Switzerland), Astellas (Tokyo, Japan), Pfizer Inc. (New York, NY, USA), KaloBios (South San Francisco, CA, USA), Clinigen (Burton-on-Trent, Staffordshire, UK), Roche (Basel, Switzerland), and Bayer (Leverkusen, Germany) and has received research grants from Sanofi Pasteur (Paris, France) and Cubist (Lexington, MA, USA). The other authors declare that they have no competing interests.
